# Maximizing safe resections: the roles of 5-aminolevulinic acid and intraoperative MR imaging in glioma surgery—review of the literature

**DOI:** 10.1007/s10143-017-0907-z

**Published:** 2017-09-18

**Authors:** Eric Suero Molina, S. Schipmann, W. Stummer

**Affiliations:** Department of Neurosurgery, University Hospital of Münster, Albert-Schweitzer-Campus 1, A1, 48149 Münster, Germany

**Keywords:** Intraoperative MRI, 5-aminolevulinic acid, Fluorescence-guided surgery, Extent of resection

## Abstract

Malignant glioma surgery involves the challenge of preserving the neurological status of patients harboring these lesions while pursuing a maximal tumor resection, which is correlated with overall and progression-free survival. Presently, several tools exist for assisting neurosurgeons in visualizing malignant tissue. Fluorescence-guided surgery (FGS) with 5-aminolevulinic acid (5-ALA) has increasingly been used during the last decade for identifying malignant glioma. Intraoperative magnetic resonance imaging (iMRI), first introduced in the mid-1990s, is being evaluated as a further tool to maximize the extent of resection. We aimed to evaluate the literature and discuss synergies and differences between FGS with 5-ALA and iMRI. We conducted and reported according to the Preferred Reporting Items for Systematic Reviews and Meta-Analysis (PRISMA) statement. After excluding non-relevant articles, 16 articles were evaluated and included in the qualitative analysis, comprising 2 (*n* = 2) reviews of the literatures, 1 (*n* = 1) book chapter, and 13 (*n* = 13) clinical articles. ALA-induced fluorescence goes beyond the borders of gadolinium contrast enhancement. Several studies stress the synergy between both tools, enabling increase in extent of resection. We point out advantages of combining both methods. iMRI, however, is not widely available, is expensive, and is not recommended as sole resection control tool in high-grade glioma. For these centers, FGS together with mapping and monitoring techniques, neuronavigation and, when needed, intraoperative ultrasound provides an excellent setting for achieving state-of-the-art gross total resection of high-grade gliomas.

## Introduction

### Extent of resection matters

Over the past years, the goal of glioma surgery has shifted from removing what is obvious to the human eye as brain tumor to what is now known to be malignant tissue assisted by technological innovations. It is now common understanding that the extent of resection (EoR) in high-grade glioma maximizes overall survival (OS) and progression-free survival (PFS) [7, 32, 41, 44, 45, 53, 54, 62], the last one being potentially jeopardized even if a small tumor remnant is left after surgery [10]. Although independent factors, i.e., age, preoperative Karnofsky performance scale (KPS), molecular markers (IDH-1 mutation, O6-methylguanin-DNA-methyltransferase, MGMT, promoter methylation), and tumor location, might play a role in influencing OS, EoR is the variable that we as neurosurgeons can influence [12, 43]. Glioma tissue infiltrates healthy brain tissue in a manner that is not perceptive to the human eye nor tangible to our hands or our instrumental extensions during surgery. This makes it challenging to identify glioma tumor tissue with white-light microscopy alone, especially at tumor margins. For this reason, several tools for the delineation of tumor tissue have been intensively explored, such as neuronavigation [84], linear array intraoperative ultrasound [12, 84], fluorescence-guided surgery (FGS) mediated by 5-aminolevulnic Acid (ALA) [75], and intraoperative magnetic resonance imaging (iMRI) [69]. The relevance, utility, additional value, and cost-effectiveness of these tools are being studied. Hence, recently, several articles have been exploring the differences and synergies between FGS and iMRI by either applying these tools simultaneously or in different cohorts during glioma surgery. We aimed to review FGS and iMRI in the context of glioma surgery and evaluate the literature for articles committed to study these tools simultaneously. Ergo, we performed a MEDLINE/PubMed search to identify relevant studies about ALA and iMRI. Our aim was to analyze synergism within both tools and compare outcomes.

## Materials and methods

### Research protocol and literature search with PRISMA

As a complementary and to guide a thorough literature search of studies where ALA and iMRI were simultaneously applied, we conducted a search and reported according to the Preferred Reporting Items for Systematic Reviews and Meta-Analysis (PRISMA) statement [46, 47]. We searched for articles published until June 2017 without neglecting any earlier publication date. The following terms were used to search for title and abstract: “ALA” and “intraoperative MRI”, “MRI” and “ALA”, “MRI” and “PPIX”, and “magnetic resonance imaging” and “aminolevulinic acid”. After excluding not relevant articles by removing duplicates as well as non-English articles and screening their titles and abstracts, we selected solely studies that evaluated FGS and iMRI simultaneously or in parallel in different cohorts. The screening of articles was performed with the help of Endnote X7 (Thompson Reuters, Carlsbad, California, USA).

## Results

**Fig. 1:**
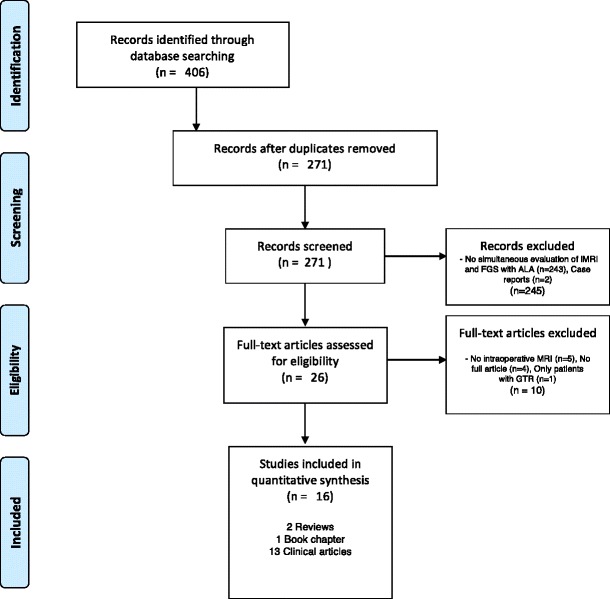
PRISMA flowchart demonstrating screening, selection, and exclusion reasons for studies

The above-mentioned search delivered 406 articles. After removing duplicates, abstracts from 280 articles were screened for relevance. After thorough evaluation and excluding articles that did not meet inclusion criteria, we identified 26 articles for full text evaluation. When relevant, we included references cited from the selected articles. We identified 16 articles to include in our qualitative synthesis. These included 1 book chapter [84], 2 reviews of the literature [3, 19], and 13 clinical studies [9, 10, 12, 22, 23, 25, 30, 50, 55, 57, 64, 83, 88], as illustrated in Fig. 1. The selected articles were published between July 2011 and June 2017 (Fig. 1). The clinical studies are summarized in Table 1.

**Table 1:** ᅟEvaluated clinical studies

Study	Year	Study type	No. of patients	ALA	Intraoperative MRI	Tumor type	Gross total resection rates with			Survival	Comment/conclusion
							ALA	MRI	Both		
Coburger et al.	2014	Prospective, single-center, histology-based assessment	45	Yes	Yes (1.5 T)	34 HGG, 11 metastatic lesions	n/a	n/a	n/a	n/a	Sensitivity for tumor detection: ALA (91%) vs. iMRI (66%); specificity for tumor detection: ALA (91%) vs. iMRI (60%)
Coburger et al.	2015	Prospective, single-center, combined with retrospective match-paired assessment	33	Yes	Yes (1.5 T)	Glioblastoma	n/a	82%	100%	PFS and OS = n.s. between groups	Significant increase in EoR when combining ALA and iMRI without higher complications rates.
Coburger et al.	2017	Prospective, single-center, histology-based assessment	33	Yes	Yes (1.5 T)	Glioblastoma	n/a	n/a	n/a	n/a	“Only 5-ALA showed a significant correlation to histopathological findings compared to iMRI and linear array intraoperative ultrasound”
Eyüpoglu et al.	2012	Prospective, single-center	37	Yes	Yes (1.5 T)	High-grade glioma	71.7%	n/a	100%*	n/a	“After initial resection with ALA, MRI assisted in finding tumor remnant which after locating it, demonstrated fluorescence. iMRI was helpful for discriminating low-grade portions of allegedly secondary high-grade tumors” (* for tumors in the vicinity of eloquent regions)
Eyüpoglu et al.	2016	Prospective (*n* = 30) and retrospective (*n* = 75), single-center	105	Yes	Yes (1.5 T)	Glioblastoma	n/a	n/a	100% (*n* = 30, supramarginal resection)	Median survival time 18.5 months (vs. 14 months in the control arm)	No iMRI or FGS in retrospective cohort
Gessler et al.	2015	Prospective, single-center	32	Yes	Yes (0.15 T)	Glioblastoma	n/a	n/a	97%	OS 80.7 weeks, PFS 61.3 weeks	Sensitivity and specificity of iMRI and 5-ALA to detect remaining tumor tissue were 75 and 100% for iMRI and 70 and 100% for 5-ALA fluorescence “in 52.6% of the cases; each one of the modalities was the only indicator of further tumor tissue in 26.3% (iMRI) or 21.1%(5-ALA) of the cases, while the other did not indicate residual tumor, when the surgeon thought to have achieved GTR already.”
Hauser et al.	2016	Prospective, single-center	14	Yes	Yes (0.15 T)	Glioblastoma	9%	n/a	82%	Mean OS 15.3 months, 6-month PFS 36.4%	iMRI demonstrated 91.6% of tumor remnant after FGS; however, only 64.3% of these were confirmed as actual tumor after histological examination
Nickel et al.	2017	Prospective, multicenter	162	Yes	Yes (n/a)	High-grade glioma	74%	94%	95%	n/a	GTR with no imaging 73% and with 5-ALA alone 74%
Quick-Weller et al.	2016	Prospective, single-center	7	Yes	Yes (0.15 T)	Recurrent glioblastoma	n/a	n/a	100%	Median OS 27.8 months, since repeat surgery 7.6 months	Absence of fluorescence in one patient. “5-ALA is a powerful surgical tool, but in the case of recurrent GBM, re-surgery should be performed under the combined help of 5-ALA and iMRI in order to achieve most radical tumor resection to prolong patients’ survival.”
Roder et al.	2014	Retrospective, single-center	117	Yes (*n* = 47)	Yes (*n* = 27; 1.5 T)	Glioblastoma	46%	74%	n/a	No statistical difference between groups	Thirty-two percent of patients in the iMRI group received ALA. Residual volume after iMRI (0.5 cm^3^) was lower than both 5-ALA (1.9 cm^3^) and white-light surgery (4.9 cm^3^). This is a historic comparison, and differences can be multifactorial.
Schatlo et al.	2015	Retrospective, single-center	200	Yes	Yes (0.15 T)	High-grade gliomas	n/a (*n* = 58)	n/a	45% (*n* = 55)	Median OS 13.8 months and PFS 7 months vs. 17.9 and 10.6 months (no iMRI vs. iMRI)	Historic comparison (2003–2011). Large groups of patients before FGS era and introduction of concomitant radio-/chemotherapy with TMZ
Tsugu et al.	2011	Retrospective, single-center	33	Yes	Yes (1.5 T)	Low- and high-grade gliomas	55%	56%*	40%	n/a	* Included 12 ALA negative low-grade gliomas
Yamada et al.	2015	Prospective, single-center	97	Yes	Yes (0.3 T)	High-grade gliomas	n/a	n/a	52%	n/a	“5-ALA-induced tissue fluorescence had 92% positive predictive value (PPV) for presence of glioma in the histopathological specimen.” “Neurochemical navigation with 5-ALA is useful adjunct during iMRI-guided resection of intracranial malignant gliomas, which allows identification of the tumor extension beyond its radiological borders.”

## Discussion

### 5-Aminolevulinic acid

5-Aminolevulinic acid, a prodrug and a precursor in heme biosynthesis, leads to accumulation of fluorescent protoporphyrin IX (PPIX) in certain glioma tumor cells, enabling their visualization with the use of commercially available microscopes equipped with a special filter system. The exact uptake mechanism of ALA in glioma cells is still not fully understood. However, it is known that ALA is selectively absorbed by tumor cells and is converted into fluorescent PPIX with the help of enzymes of the heme biosynthesis [14]. First introduced in 1998 [78], ALA has been extensively investigated in and ex vivo, finally obtaining approval in Europe and many further countries after a randomized phase III trial [75]. Recently, ALA was also approved for fluorescence-guided resections of gliomas in the USA.

**Fig. 2:**
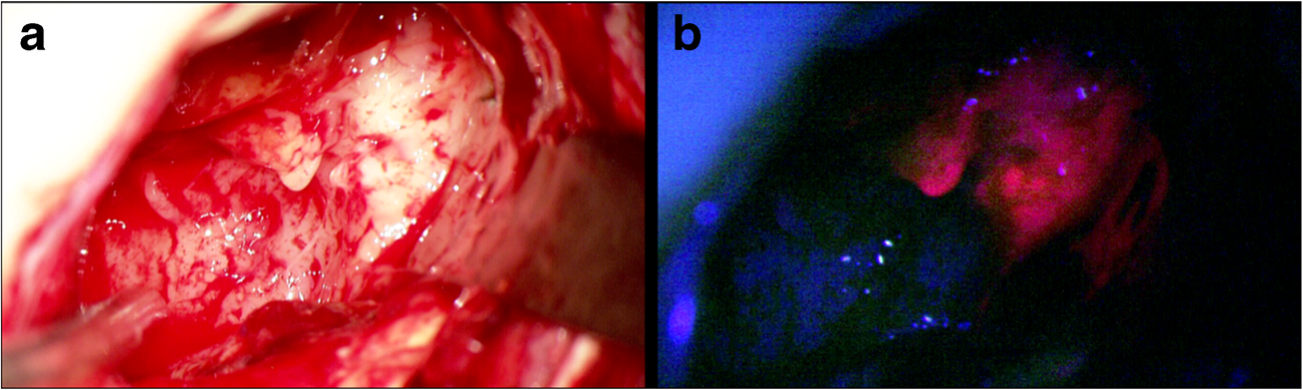
Fluorescence-guided surgery with ALA in a patient harboring a glioblastoma demonstrating the intraoperative view of a glioblastoma under **a** white-light microscopy and **b** PPIX fluorescence with BLUE 400 filter (Zeiss, Oberkochen, Germany)

A high selectivity of malignant glioma cells for PPIX fluorescence has been observed in several studies presented over the last decades, and normal brain tissue does not appear to induce PPIX expression after ALA administration [9, 17, 20, 31, 34, 74, 79]. To date, ALA is administered as an oral solution at a dose of 20 mg/kg body weight 4 hours before anesthesia induction [75]. Not only from surgical experience, but also from ex vivo studies, we know that peak fluorescence will be expected around 6–8 h after administration [77]. A recent report explored the low toxicology and the safety profile of ALA [82]. Besides rare transient liver enzyme elevation and known light sensitivity of the skin 24 h after administration, ALA appears to be safe. So far, more than 30,000 patients in over 30 countries have been treated with ALA-induced fluorescence [72]. In order to visualize PPIX fluorescence, a modern microscope equipped with a blue/violet light with a wave length of 375–440 nm together with an emission filter is needed, enabling the visualization of red fluorescence at a first peak of 635 nm and a second peak at 704 nm (Fig. 2).

Green fluorescence, which is the way tissue autofluorescence appears, will enable background information to allow visualization of surrounding tissue [76]. This information, however, can sometimes be too weak for adequate discrimination of the surgical field, requiring surgeons to alternate between white-light microscopy and blue fluorescence, e.g., for hemostasis. When tumor cell density in tumor tissue is above 10%, PPIX fluorescence visualization will be expected [79]. However, in recurrent gliomas, some caveats merit mention when operating with FGS and ALA, since altered brain tissue and gliosis areas along with reactive astrocytes might induce ALA uptake and provide PPIX fluorescence [48]. Therefore, concerns have been raised regarding the specificity of PPIX fluorescence in recurrent gliomas [14]. In a recent study, however, the diagnostic accuracy of fluorescent tumor tissue did not significantly differ between primary and recurrent glioblastomas [88]. Furthermore, authors have reported that in certain cases, PPIX fluorescence can help differentiate recurrent tumor from scar tissue [10]. Another important pitfall is that fluorescence can be hidden in the tumor cavity, e.g., when craniotomy is too small, or behind a thin layer of healthy brain or blood [23, 25]. FGS with ALA 2-D is an imaging surface tool, with which depth can limit visualization of tumor tissue. Anatomical landmarks—as well as further tools such as intraoperative ultrasound, neuronavigation, and iMRI—can be applied when available, to avoid missing tumor tissue.

### Intraoperative MRI

Since their first introduction in the mid-1990s [6], low- (0.15–0.5 T) and high-field iMRI (1.5–3.0 T) are being investigated for resection control in glioma surgery [9, 13, 37, 49, 69, 70]. iMRI can provide relevant real-time imaging and feedback on the resection status during surgery. It allows to update the neuronavigation system which can increase accuracy during resection, making brain shift after initial resection a lesser problem [28, 29, 37, 38]. Nimsky et al. [51] evaluated iMRI in the context with intraoperative neuronavigation and demonstrated the advantages of combining both tools.

Senft et al. performed the first randomized controlled trial evaluating iMRI in glioma surgery and demonstrated a gross total resection (GTR) rate of 96% in the iMRI arm, compared to 68% in the control group with conventional microsurgery [69]. Several studies have evaluated the clinical value of low-field iMRI [25, 30, 33]. Extension in EoR and PFS was demonstrated by Senft et al. in their iMRI group using a low-field iMRI (0.15 T), whereas Coburger et al. demonstrated the benefit of high-field (1.5 T) vs. low-field (0.15) iMRI regarding GTR; however, it did not affect PFS [11]. Bergsneider et al. [5] found no statistically significant difference in the EoR after retrospectively evaluating patients operated either with a 0.15 or 1.5 T iMRI. However, as evaluated in a recent report, it can falsely demonstrate gadolinium (Gd) enhancement and lead to low predictive value (64.3%) for iMRI-guided tumor recognition and potentially to extended resection of healthy tissue [16, 30]. Hatiboglu et al. demonstrated an EoR of up to 96% with the help of iMRI in glioma surgery [29], whereas Wirtz et al. showed that in 62% of patients, 0.2 T iMRI was helpful in finding tumor remnants after initial tumor resection [87]. A large, recently published series of 170 glioblastomas operated with iMRI demonstrated a significant impact of EoR to OS [13]. This study demonstrates the importance of a multimodal approach. In this case, the authors stressed the importance of performing neurophysiological monitoring whenever eloquent tissue was expected during surgery.

**Fig. 3:**
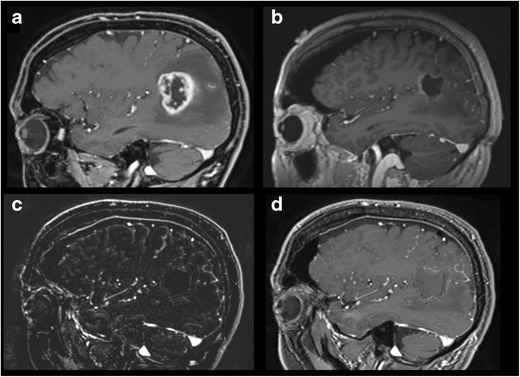
Pre-, intra-, and postoperative MRI of a patient harboring a high-grade glioma. **a** Pre-, **b** intra-, and **c**, **d** postoperative contrast-enhanced T1-weighted MRI of a patient harboring a glioblastoma. **c** demonstrates the subtraction imaging after Gd application. Postoperative MRI was performed within 48 h after surgery. Intraoperative imaging with iMRI, which demonstrated what appears to be a remnant rostral of the tumor cavity, was, moreover, revealed as Gd-leakage. Biopsy of this region demonstrated no tumor tissue, and Gd-enhanced postoperative MRI and subtraction imaging did not confirm tumor residual (**c**, **d**) (Courtesy of Dr. Ricardo Diez-Valle, Department of Neurosurgery, Clinica Universidad de Navarra, Navarre, Spain)

To mention some limitations, iMRI is of high cost. Furthermore, surgery and anesthesia time will be relevantly increased (~ 1 h), since a pause is required to perform and evaluate images [23, 69]. Additionally, frequent application of Gd during surgery will lead to extravasation into the resection cavity, making interpretation of imaging challenging (Fig. 3) [1].

### 5-Aminolevulinic acid-induced fluorescence vs. gadolinium contrast enhancement in MRI

How accurate is the relationship between PPIX fluorescence and Gd contrast enhancement in MRI? In an early series of the FGS era by Stummer et al. in the year of 2000, 17 from 52 patients did not demonstrate residual tumor in early postoperative MRI (1.5 T), even though residual fluorescence remained after resection due to the eloquence of these regions [74]. Another study discussed the fact that PPIX fluorescence is, based on histopathological assessment, superior to Gd contrast enhancement in MRI with a significantly higher specificity and sensitivity for glioma tumor detection [9]. This indicates that fluorescent tissue, i.e., malignant tumor tissue, goes beyond the Gd uptake demonstrated in MRI [2, 10, 58, 67]. In this context, one group discussed the fact that fluorescent tissue volume doubles the size of contrast enhancement on MRI [67]. Furthermore, Roessler et al. [58] demonstrated fluoro-ethyl-positron emission tomography (FET-PET) hypermetabolism zone to be smaller than the fluorescent tissue, while others state that PPIX fluorescence matches preoperative FET-PET tracer uptake [71]. Corburger et al. noticed a higher sensitivity for tumor detection at the margins of tumor infiltration with ALA compared to iMRI when simultaneously using them (Table 1) [10]. Moreover, it has been shown that malignant glioma infiltration can exceed Gd contrast enhancement by 6–14 mm as demonstrated in a comparison of preoperative MRI and post mortem neuropathological assessment [89]. Another group, Aldave et al. [2], concluded in a cohort of 50 patients that those without residual fluorescence and overall no residual Gd enhancement in early postoperative MRI lived 10 months longer compared to those with residual fluorescence. It must be mentioned that MRI might be limited by the quantity of blood brain breakdown or tumor cluster size, making PPIX fluorescence superior regarding diagnostic accuracy [61].

Gd contrast enhancement in MRI will demonstrate predominantly blood brain barrier breakdown, which is a part of but might not demonstrate the complete tumor, whereas ALA will be metabolized specifically by tumor cells and is not completely correlated with blood brain barrier breakdown [74]. This is an important principle that might help us understand the differences between both tools. As mentioned above and to indicate another limitation, ALA will demonstrate fluorescence starting from a tumor cell density of 10%.

Concluding, the reviewed articles demonstrate important synergies between both tools. However, evidence level for simultaneously applying both tools is still low. iMRI alone might not provide enough intraoperative tumor identification, and it lacks the possibility of live guidance. Hence, it should be evaluated in further studies.

### Resection rates

If we know that available tools can improve the extent of resection without harming eloquent tissue and enabling state-of-the-art glioma resection, then why not use them as they are available to us?

Gross total resection, as to date the main goal of glioma surgery, is defined as no residual Gd contrast enhancement in early postoperative MRI [44, 62]. Residual cells, at tumor margin, appear to be of relevant importance for survival [26]. Sanai et al. demonstrated a threshold of > 78% EoR to have the highest impact on patients’ survival and recommended to use this knowledge for surgical decision making, e.g., in tumors where subtotal resection is planned due to eloquence [63]. Up to now, level I evidence exists only for FGS with ALA for improving EoR and OS in patients harboring malignant gliomas [75], whereas level II evidence has been provided for iMRI [69]. In spite of the fact that the first reported complete resection rates with ALA and FGS in the randomized phase III trial of 65% are at present considered low, we have to remember that they were still twice as high as when operating under white-light microscopy [75] and all series with data on resection rates, especially if single-armed and retrospective, will depend very strongly on case selection and the respective surgeon. So far, new technological advantages, i.e., neuronavigation, mapping and monitoring, and intraoperative ultrasound, have been developed and are frequently available, helping increase complete resection rates [81]. Recent studies of patients operated with FGS and ALA report a resection rate close to 90% when mapping and monitoring techniques are applied (Table 2) [15, 17, 66, 68, 73], shifting the reason for incomplete resections to be eloquence of the region rather than unawareness of the presence of the malignant tissue. In a recent report, ALA-induced fluorescence demonstrated a positive predictive value of over 95% [27].

**Table 2:** Articles simultaneously researching iMRI and FGS with ALA in glioma surgery

Study	Stummer et al.	Stummer et al.	Diez Valle et al.	Schucht et al.	Della Puppa et al.	Schucht et al.
Year	2000	2006	2011	2012	2013	2014
No. of patients	50	135	36	103	25	67
Study design	Prospective, single-center	Prospective, multicenter two-arm randomized	Prospective, single-center	Prospective, single-center	Prospective, single-center	Prospective, single-center
Eloquent region	Eloquent and non-eloquent	Eloquent and non-eloquent	Eloquent and non-eloquent	Eloquent and non-eloquent	Eloquent and non-eloquent	Solely eloquent (motor)
Monitoring and mapping	No	No	Yes	Yes	Yes	Yes
Resection rate	65%	65%	83.3%	96%	80%	76%

Supra-marginal resection is being discussed as potentially feasible for malignant gliomas [42], predominantly in the context of low-grade gliomas [18, 90]. Thus, Eyüpoglu et al. evaluated a prospective supra-marginally resected collective of 30 patients with glioblastoma operated with both FGS with ALA and iMRI and performed a historic comparison to a retrospectively analyzed cohort (*n* = 75) operated with neuronavigation (Table 1) [22]. The authors found a significant extension of median overall survival (18.5 vs. 14 months) in the prospective arm. Nevertheless, they included patients in the control arm before important developments became standard, such as adjuvant temozolomide treatment concomitant to radiotherapy [80]. Therefore, such improvements may have multifactorial etiology. The same authors recognized the advantage of iMRI in cases where initially, FGS was performed, and in absence of obvious fluorescence iMRI indicated tumor remnants that led again to finding fluorescent tissue which was first overlooked; for instance, being hidden in parts of the cavity that were not easily accessed (Table 1).

### Low-grade gliomas

Present evidence indicates that the extent of resection in low-grade gliomas will reduce the risk of recurrence and increase overall survival [8, 18].

We are now well aware that fairly 20% of non-enhancing low- and high-grade tumors will demonstrate PPIX fluorescence when applied [21, 35, 52, 71, 73, 85, 86]. Furthermore, in 44–55% of cases where lesions in the preoperative MRI are suggestive of low-grade glioma, an anaplastic focus could still be discovered [39, 71]. Our group evaluated preoperative factors for predicting fluorescence in gliomas. We concluded that age, tumor, volume, and ^18^F-FET-PET uptake ratio > 1.85 are significant factors for predicting fluorescence [35].

However, due to the high number of non-fluorescing low-grade gliomas, relying on ALA alone for GTR will not meet the expectations of the surgeon. For these cases, iMRI is a helpful tool to achieve maximal surgical outcome [83]. Also, for low-grade portions of high-grade gliomas or satellite lesions, iMRI can help discover these elements to achieve GTR. In contrast, Senft et al. reported not having achieved GTR in a large portion of low-grade glioma using a low-field iMRI that has a low anatomical resolution [70]. Nevertheless, Hirschl et al. [33] found low-field iMRI to be feasible for detecting residual low-grade glioma (sensitivity 82%, specificity 95%). Thus, studies applying high-field iMRI report much higher resection rates for non-fluorescing and enhancing gliomas [29, 83], which is probably more suitable for low-grade glioma surgery. However, data regarding the impact of OS associated with EoR in low-grade gliomas remains scarce.

For centers without iMRI, the combination of intraoperative ultrasonography, together with adequate mapping and monitoring techniques and neuronavigation, can be a reliable combination for safe resections while attempting maximal radicality [12, 19, 84]. ALA is to date not recommended to be of standard use in low-grade glioma surgery.

### Cost-effectiveness

Since how we evaluate treatments is changing over time, i.e., from a more economically focused volume-based health care to an outcome- and value-driven healthcare delivery, cost-effectiveness analyses are becoming more and more important. Outcome is now often being measured in quality-adjusted life years (QALYs), and together with costs, ratios are being built, in order to evaluate the costs of healthcare delivery and facilitate decision-making when implementing new interventions. For this purpose, quality indicators are emerging in each medical field to help define treatments’ outcome [65]. Because of the novelty of this transition, scientific data remains to date scarce. A study evaluating effectiveness and cost-effectiveness from different intraoperative imaging modalities, i.e., FGS (ALA and fluorescein), ultrasound and iMRI, was recently published [19]. The authors calculated a cost-effectiveness ratio of $1784 for FGS with ALA vs. $3625 for iMRI. Additional cost per QALY gained amounted $16,218 for FGS with ALA and $32,955 for iMRI. Despite the authors basing some of their calculations on older articles, it is an important evaluation on which future studies can build upon. For instance, in the UK, the National Health Service (NHS) will in general fund treatments between £20,000 and £30,000 per QALY gained. Hence, interventions costing less than £20,000 are cost-effective. In the same manner, willingness-to-pay threshold can be up to $50,000 for malignant glioma, as it has been calculated in the USA [56]. According to Eljamel et al., iMRI is more expensive than ALA, yet it is below the quite high willingness-to-pay threshold. Furthermore, Esteves et al. performed a pilot cost-effectiveness analysis with a Markov model in the Portuguese healthcare system of FGS with ALA vs. conventional white-light surgery. The authors calculated the cost per QALY gained with FGS and ALA to be around €9100. Nevertheless, costs depend on the country and healthcare system. It is important to obtain more data on cost-effectiveness in order to support neurosurgeons in decision-making toward establishing new interventions, tools, drugs, or procedures. Consequently, further studies are needed. From the available data, iMRI was only stated to be cost-effective in the USA; for other countries, the high costs per QALY are over the willingness-to-pay-threshold.

### Present era in the surgical treatment of gliomas

Why spend effort in trying to demonstrate that one tool is better than the other, instead of trying to find synergism between them? As stated above, it is known that the extent of resection maximizes not only progression-free survival but also overall survival [7, 32, 41, 44, 45, 53, 54, 62]. Consequently, as neurosurgeons, we should use all available tools to increase the impact of our surgery [4]. A multimodal approach, however, is difficult to evaluate. In such setting, it is a challenge to dissect the independent individual clinical value of each tool applied. Only a few of the available articles performed multivariate analyses tackling this problem [3]. Another important fact is that the available series often included retrospective analyses of patients treated before the combined radio-/chemotherapy with temozolomide was introduced as an adjuvant treatment for glioblastoma, as described by Stupp et al. [80], which significantly increased PFS and OS. Other authors have already recognized this problem and are offering more recent data in the new era of glioma treatment [13].

Despite access to the discussed tools, neurosurgeons will still require a profound understanding of neuroanatomy and function localization with mapping and monitoring techniques. Resection is limited by eloquent regions, as in regions with motor and language functions, and function preservation should be prioritized against radical tumor removal to keep patients’ quality of life and independence as high as possible [50]. By cortical and subcortical stimulations, the surgeon can identify functional pathways that should be avoided during surgery [84]. Intraoperative neurophysiologic monitoring (IOM) consists of “mapping,” with the surgeon identifying and defining language and sensory and motor areas with the aid of cortex stimulation during surgery, and “monitoring”, defined by the continuous assessment of the functional integrity of neural pathways [59, 60]. Different types of evoked potentials can be assessed, i.e., motor-evoked potentials, somatosensory-evoked potentials and, more rarely, visual-evoked potentials [59].

In a prospective trial, Kombos and colleagues evaluated both tumors in non-eloquent areas without IOM and tumors within or next to eloquent areas with IOM and found no significant difference in EoR without jeopardizing neurological outcome. This important finding suggests that equally aggressive surgical removals of eloquent tumors are warranted under the right settings [36].

The undisputed advantage of 5-aminolevulinic acid and fluorescence-guided surgery is the real-time information provided during the actual surgery. Nevertheless, efforts should be woven into finding synergism between these tools. Hence, both methods can complementarily improve the extent of resection in malignant gliomas [25]. Resources should be used as available, since all of them, to a certain level, increase patients’ safety while maximizing tumor resection [4, 24, 40].

### Summary

ALA-induced fluorescence goes beyond the borders of Gd contrast enhancement. For identifying tumor tissue, present evidence suggests FGS with ALA to be superior for intraoperative tumor identification and iMRI should only be used in combination with FGS in HGG. On the other hand, iMRI can help overcome FGS weakness regarding depth and tumor residual due to limited view of the tumor tissue, i.e., after too small craniotomies. The combination of a 2-D imaging surface tool, as it is with FGS with ALA, together with the information of intraoperative 3-D imaging, as it is with iMRI, can help achieve high rates of GTR. For LGG, iMRI could be relevant to increase EoR, since FGS with ALA is not useful in most cases. However, iMRI is an expensive adjunct and is not everywhere available. For these centers, we believe that FGS together with mapping and monitoring techniques, neuronavigation, and, when needed, intraoperative ultrasound provides an excellent setting for achieving state-of-the-art GTR of malignant gliomas.

## References

[1] Albert FK, Wirtz CR, Tronnier VM, Hamer J, Bonsanto MM, Staubert A, Knauth M, Kunze S, Hellwig D, Bauer BL (1998). Intraoperative diagnostic and interventional MRI in neurosurgery: first experience with an “open MR” system. Minimally invasive techniques for neurosurgery: current status and future perspectives.

[2] Aldave G, Tejada S, Pay E, Marigil M, Bejarano B, Idoate MA, Diez-Valle R (2013). Prognostic value of residual fluorescent tissue in glioblastoma patients after gross total resection in 5-aminolevulinic acid-guided surgery. Neurosurgery.

[3] Barbosa BJ, Mariano ED, Batista CM, Marie SK, Teixeira MJ, Pereira CU, Tatagiba MS, Lepski GA (2015). Intraoperative assistive technologies and extent of resection in glioma surgery: a systematic review of prospective controlled studies. Neurosurg Rev.

[4] Berger MS (2014). Use of 5-aminolevulinic acid helps see the way beyond MRI. Neurosurg Focus.

[5] Bergsneider M, Sehati N, Villablanca P, McArthur DL, Becker DP, Liau LM (2005). Mahaley clinical research award: extent of glioma resection using low-field (0.2 T) versus high-field (1.5 T) intraoperative MRI and image-guided frameless neuronavigation. Clin Neurosurg.

[6] Black PM, Moriarty T, Alexander E, Stieg P, Woodard EJ, Gleason PL, Martin CH, Kikinis R, Schwartz RB, Jolesz FA (1997). Development and implementation of intraoperative magnetic resonance imaging and its neurosurgical applications. Neurosurgery.

[7] Chang EF, Clark A, Smith JS, Polley MY, Chang SM, Barbaro NM, Parsa AT, McDermott MW, Berger MS (2011). Functional mapping-guided resection of low-grade gliomas in eloquent areas of the brain: improvement of long-term survival. Clinical article. J Neurosurg.

[8] Claus EB, Horlacher A, Hsu L, Schwartz RB, Dello-Iacono D, Talos F, Jolesz FA, Black PM (2005). Survival rates in patients with low-grade glioma after intraoperative magnetic resonance image guidance. Cancer.

[9] Coburger J, Engelke J, Scheuerle A, Thal DR, Hlavac M, Wirtz CR, Konig R (2014). Tumor detection with 5-aminolevulinic acid fluorescence and Gd-DTPA-enhanced intraoperative MRI at the border of contrast-enhancing lesions: a prospective study based on histopathological assessment. Neurosurg Focus.

[10] Coburger J, Hagel V, Wirtz CR, Konig R (2015). Surgery for glioblastoma: impact of the combined use of 5-aminolevulinic acid and intraoperative MRI on extent of resection and survival. PLoS One.

[11] Coburger J, Merkel A, Scherer M, Schwartz F, Gessler F, Roder C, Pala A, Konig R, Bullinger L, Nagel G, Jungk C, Bisdas S, Nabavi A, Ganslandt O, Seifert V, Tatagiba M, Senft C, Mehdorn M, Unterberg AW, Rossler K, Wirtz CR (2016). Low-grade glioma surgery in intraoperative magnetic resonance imaging: results of a multicenter retrospective assessment of the German study group for intraoperative magnetic resonance imaging. Neurosurgery.

[12] Coburger J, Scheuerle A, Pala A, Thal D, Wirtz CR, Konig R (2017) Histopathological insights on imaging results of intraoperative magnetic resonance imaging, 5-aminolevulinic acid, and intraoperative ultrasound in glioblastoma surgery. Neurosurgery. 10.1093/neuros/nyw143 10.1093/neuros/nyw143 28204539

[13] Coburger J, Wirtz CR, Konig RW (2017). Impact of extent of resection and recurrent surgery on clinical outcome and overall survival in a consecutive series of 170 patients for glioblastoma in intraoperative high field magnetic resonance imaging. J Neurosurg Sci.

[14] Colditz MJ, Leyen K, Jeffree RL (2012). Aminolevulinic acid (ALA)-protoporphyrin IX fluorescence guided tumour resection. Part 2: theoretical, biochemical and practical aspects. J Clin Neurosci.

[15] Della Puppa A, De Pellegrin S, d'Avella E, Gioffre G, Rossetto M, Gerardi A, Lombardi G, Manara R, Munari M, Saladini M, Scienza R (2013). 5-Aminolevulinic acid (5-ALA) fluorescence guided surgery of high-grade gliomas in eloquent areas assisted by functional mapping. Our experience and review of the literature. Acta Neurochir (Wien).

[16] Della Puppa A, Rustemi O, Rampazzo E, Persano L (2017). Letter: combining 5-aminolevulinic acid fluorescence and intraoperative magnetic resonance imaging in glioblastoma surgery: a histology-based evaluation. Neurosurgery.

[17] Diez Valle R, Tejada Solis S, Idoate Gastearena MA, Garcia de Eulate R, Dominguez Echavarri P, Aristu Mendiroz J (2011). Surgery guided by 5-aminolevulinic fluorescence in glioblastoma: volumetric analysis of extent of resection in single-center experience. J Neuro-Oncol.

[18] Duffau H (2012). Awake surgery for incidental WHO grade II gliomas involving eloquent areas. Acta Neurochir.

[19] Eljamel MS, Mahboob SO (2016). The effectiveness and cost-effectiveness of intraoperative imaging in high-grade glioma resection; a comparative review of intraoperative ALA, fluorescein, ultrasound and MRI. Photodiagn Photodyn Ther.

[20] Eljamel S (2015). 5-ALA fluorescence image guided resection of glioblastoma multiforme: a meta-analysis of the literature. Int J Mol Sci.

[21] Ewelt C, Floeth FW, Felsberg J, Steiger HJ, Sabel M, Langen KJ, Stoffels G, Stummer W (2011). Finding the anaplastic focus in diffuse gliomas: the value of Gd-DTPA enhanced MRI, FET-PET, and intraoperative, ALA-derived tissue fluorescence. Clin Neurol Neurosurg.

[22] Eyupoglu IY, Hore N, Merkel A, Buslei R, Buchfelder M, Savaskan N (2016). Supra-complete surgery via dual intraoperative visualization approach (DiVA) prolongs patient survival in glioblastoma. Oncotarget.

[23] Eyupoglu IY, Hore N, Savaskan NE, Grummich P, Roessler K, Buchfelder M, Ganslandt O (2012). Improving the extent of malignant glioma resection by dual intraoperative visualization approach. PLoS One.

[24] Feigl GC, Ritz R, Moraes M, Klein J, Ramina K, Gharabaghi A, Krischek B, Danz S, Bornemann A, Liebsch M, Tatagiba MS (2010). Resection of malignant brain tumors in eloquent cortical areas: a new multimodal approach combining 5-aminolevulinic acid and intraoperative monitoring. J Neurosurg.

[25] Gessler F, Forster MT, Duetzmann S, Mittelbronn M, Hattingen E, Franz K, Seifert V, Senft C (2015). Combination of intraoperative magnetic resonance imaging and intraoperative fluorescence to enhance the resection of contrast enhancing gliomas. Neurosurgery.

[26] Glas M, Rath BH, Simon M, Reinartz R, Schramme A, Trageser D, Eisenreich R, Leinhaas A, Keller M, Schildhaus HU, Garbe S, Steinfarz B, Pietsch T, Steindler DA, Schramm J, Herrlinger U, Brustle O, Scheffler B (2010). Residual tumor cells are unique cellular targets in glioblastoma. Ann Neurol.

[27] Hadjipanayis CG, Widhalm G, Stummer W (2015). What is the surgical benefit of utilizing 5-aminolevulinic acid for fluorescence-guided surgery of malignant gliomas?. Neurosurgery.

[28] Hall WA, Galicich W, Bergman T, Truwit CL (2006). 3-Tesla intraoperative MR imaging for neurosurgery. J Neuro-Oncol.

[29] Hatiboglu MA, Weinberg JS, Suki D, Rao G, Prabhu SS, Shah K, Jackson E, Sawaya R (2009). Impact of intraoperative high-field magnetic resonance imaging guidance on glioma surgery: a prospective volumetric analysis. Neurosurgery.

[30] Hauser SB, Kockro RA, Actor B, Sarnthein J, Bernays RL (2016). Combining 5-aminolevulinic acid fluorescence and intraoperative magnetic resonance imaging in glioblastoma surgery: a histology-based evaluation. Neurosurgery.

[31] Hefti M, von Campe G, Moschopulos M, Siegner A, Looser H, Landolt H (2008). 5-Aminolevulinic acid induced protoporphyrin IX fluorescence in high-grade glioma surgery: a one-year experience at a single institutuion. Swiss Med Wkly.

[32] Hess KR (1999). Extent of resection as a prognostic variable in the treatment of gliomas. J Neuro-Oncol.

[33] Hirschl RA, Wilson J, Miller B, Bergese S, Chiocca E (2009). The predictive value of low-field strength magnetic resonance imaging for intraoperative residual tumor detection. Clinical article. J Neurosurg.

[34] Idoate MA, Diez Valle R, Echeveste J, Tejada S (2011). Pathological characterization of the glioblastoma border as shown during surgery using 5-aminolevulinic acid-induced fluorescence. Neuropathology.

[35] Jaber M, Wolfer J, Ewelt C, Holling M, Hasselblatt M, Niederstadt T, Zoubi T, Weckesser M, Stummer W (2016). The value of 5-aminolevulinic acid in low-grade gliomas and high-grade gliomas lacking glioblastoma imaging features: an analysis based on fluorescence, magnetic resonance imaging, 18F-fluoroethyl tyrosine positron emission tomography, and tumor molecular factors. Neurosurgery.

[36] Kombos T, Picht T, Derdilopoulos A, Suess O (2009). Impact of intraoperative neurophysiological monitoring on surgery of high-grade gliomas. J Clin Neurophysiol.

[37] Kubben PL, ter Meulen KJ, Schijns OE, ter Laak-Poort MP, van Overbeeke JJ, van Santbrink H (2011). Intraoperative MRI-guided resection of glioblastoma multiforme: a systematic review. Lancet Oncol.

[38] Kuhnt D, Ganslandt O, Schlaffer SM, Buchfelder M, Nimsky C (2011). Quantification of glioma removal by intraoperative high-field magnetic resonance imaging: an update. Neurosurgery.

[39] Kunz M, Thon N, Eigenbrod S, Hartmann C, Egensperger R, Herms J, Geisler J, la Fougere C, Lutz J, Linn J, Kreth S, von Deimling A, Tonn JC, Kretzschmar HA, Popperl G, Kreth FW (2011). Hot spots in dynamic (18)FET-PET delineate malignant tumor parts within suspected WHO grade II gliomas. Neuro-Oncology.

[40] Lacroix M, Abi-Said D, Fourney DR, Gokaslan ZL, Shi W, DeMonte F, Lang FF, McCutcheon IE, Hassenbusch SJ, Holland E, Hess K, Michael C, Miller D, Sawaya R (2001). A multivariate analysis of 416 patients with glioblastoma multiforme: prognosis, extent of resection, and survival. J Neurosurg.

[41] Lawrence YR, Mishra MV, Werner-Wasik M, Andrews DW, Showalter TN, Glass J, Shen X, Symon Z, Dicker AP (2012). Improving prognosis of glioblastoma in the 21st century: who has benefited most?. Cancer.

[42] Li YM, Suki D, Hess K, Sawaya R (2016). The influence of maximum safe resection of glioblastoma on survival in 1229 patients: can we do better than gross-total resection?. J Neurosurg.

[43] Marko NF, Weil RJ, Schroeder JL, Lang FF, Suki D, Sawaya RE (2014). Extent of resection of glioblastoma revisited: personalized survival modeling facilitates more accurate survival prediction and supports a maximum-safe-resection approach to surgery. J Clin Oncol.

[44] McGirt MJ, Chaichana KL, Attenello FJ, Weingart JD, Than K, Burger PC, Olivi A, Brem H, Quinones-Hinojosa A (2008). Extent of surgical resection is independently associated with survival in patients with hemispheric infiltrating low-grade gliomas. Neurosurgery.

[45] McGirt MJ, Chaichana KL, Gathinji M, Attenello FJ, Than K, Olivi A, Weingart JD, Brem H, Quinones-Hinojosa AR (2009). Independent association of extent of resection with survival in patients with malignant brain astrocytoma. J Neurosurg.

[46] Moher D, Liberati A, Tetzlaff J, Altman DG, Group P (2009). Preferred reporting items for systematic reviews and meta-analyses: the PRISMA statement. BMJ.

[47] Moher D, Liberati A, Tetzlaff J, Altman DG, Group P (2010). Preferred reporting items for systematic reviews and meta-analyses: the PRISMA statement. Int J Surg.

[48] Nabavi A, Thurm H, Zountsas B, Pietsch T, Lanfermann H, Pichlmeier U, Mehdorn M, Group ALARGS (2009). Five-aminolevulinic acid for fluorescence-guided resection of recurrent malignant gliomas: a phase ii study. Neurosurgery.

[49] Napolitano M, Vaz G, Lawson TM, Docquier MA, van Maanen A, Duprez T, Raftopoulos C (2014). Glioblastoma surgery with and without intraoperative MRI at 3.0T. Neurochirurgie.

[50] Nickel K, Renovanz M, Konig J, Stockelmaier L, Hickmann AK, Nadji-Ohl M, Engelke J, Weimann E, Freudenstein D, Ganslandt O, Bullinger L, Wirtz CR, Coburger J (2017) The patients’ view: impact of the extent of resection, intraoperative imaging, and awake surgery on health-related quality of life in high-grade glioma patients-results of a multicenter cross-sectional study. Neurosurg Rev. 10.1007/s10143-017-0836-x 10.1007/s10143-017-0836-x 28265818

[51] Nimsky C, Ganslandt O, Buchfelder M, Fahlbusch R (2006). Intraoperative visualization for resection of gliomas: the role of functional neuronavigation and intraoperative 1.5 T MRI. Neurol Res.

[52] Nishikawa R (2011). Fluorescence illuminates the way. Neuro-Oncology.

[53] Oszvald A, Guresir E, Setzer M, Vatter H, Senft C, Seifert V, Franz K (2012). Glioblastoma therapy in the elderly and the importance of the extent of resection regardless of age. J Neurosurg.

[54] Pichlmeier U, Bink A, Schackert G, Stummer W, Group ALAGS (2008). Resection and survival in glioblastoma multiforme: an RTOG recursive partitioning analysis of ALA study patients. Neuro-Oncology.

[55] Quick-Weller J, Lescher S, Forster MT, Konczalla J, Seifert V, Senft C (2016). Combination of 5-ALA and iMRI in re-resection of recurrent glioblastoma. Br J Neurosurg.

[56] Raizer JJ, Fitzner KA, Jacobs DI, Bennett CL, Liebling DB, Luu TH, Trifilio SM, Grimm SA, Fisher MJ, Haleem MS, Ray PS, McKoy JM, DeBoer R, Tulas KM, Deeb M, McKoy JM (2015). Economics of malignant gliomas: a critical review. J Oncol Pract.

[57] Roder C, Bisdas S, Ebner FH, Honegger J, Naegele T, Ernemann U, Tatagiba M (2014). Maximizing the extent of resection and survival benefit of patients in glioblastoma surgery: high-field iMRI versus conventional and 5-ALA-assisted surgery. Eur J Surg Oncol.

[58] Roessler K, Becherer A, Donat M, Cejna M, Zachenhofer I (2012). Intraoperative tissue fluorescence using 5-aminolevolinic acid (5-ALA) is more sensitive than contrast MRI or amino acid positron emission tomography ((18)F-FET PET) in glioblastoma surgery. Neurol Res.

[59] Saito T, Muragaki Y, Maruyama T, Tamura M, Nitta M, Okada Y (2015). Intraoperative functional mapping and monitoring during glioma surgery. Neurol Med Chir (Tokyo).

[60] Sala F, Lanteri P (2003). Brain surgery in motor areas: the invaluable assistance of intraoperative neurophysiological monitoring. J Neurosurg Sci.

[61] Samkoe KS, Gibbs-Strauss SL, Yang HH, Khan Hekmatyar S, Jack Hoopes P, O'Hara JA, Kauppinen RA, Pogue BW (2011). Protoporphyrin IX fluorescence contrast in invasive glioblastomas is linearly correlated with Gd enhanced magnetic resonance image contrast but has higher diagnostic accuracy. J Biomed Opt.

[62] Sanai N, Berger MS (2011). Extent of resection influences outcomes for patients with gliomas. Rev Neurol (Paris).

[63] Sanai N, Polley MY, McDermott MW, Parsa AT, Berger MS (2011). An extent of resection threshold for newly diagnosed glioblastomas. J Neurosurg.

[64] Schatlo B, Fandino J, Smoll NR, Wetzel O, Remonda L, Marbacher S, Perrig W, Landolt H, Fathi AR (2015). Outcomes after combined use of intraoperative MRI and 5-aminolevulinic acid in high-grade glioma surgery. Neuro-Oncology.

[65] Schipmann S, Schwake M, Suero Molina E, Roeder N, Steudel WI, Warneke N, Stummer W (2017) Quality indicators in cranial neurosurgery: which are presently substantiated?—a systematic review. World Neurosurg. 10.1016/j.wneu.2017.03.111 10.1016/j.wneu.2017.03.111 28465269

[66] Schucht P, Beck J, Abu-Isa J, Andereggen L, Murek M, Seidel K, Stieglitz L, Raabe A (2012). Gross total resection rates in contemporary glioblastoma surgery: results of an institutional protocol combining 5-aminolevulinic acid intraoperative fluorescence imaging and brain mapping. Neurosurgery.

[67] Schucht P, Knittel S, Slotboom J, Seidel K, Murek M, Jilch A, Raabe A, Beck J (2014). 5-ALA complete resections go beyond MR contrast enhancement: shift corrected volumetric analysis of the extent of resection in surgery for glioblastoma. Acta Neurochir.

[68] Schucht P, Seidel K, Beck J, Murek M, Jilch A, Wiest R, Fung C, Raabe A (2014). Intraoperative monopolar mapping during 5-ALA-guided resections of glioblastomas adjacent to motor eloquent areas: evaluation of resection rates and neurological outcome. Neurosurg Focus.

[69] Senft C, Bink A, Franz K, Vatter H, Gasser T, Seifert V (2011). Intraoperative MRI guidance and extent of resection in glioma surgery: a randomised, controlled trial. Lancet Oncol.

[70] Senft C, Seifert V, Hermann E, Franz K, Gasser T (2008). Usefulness of intraoperative ultra low-field magnetic resonance imaging in glioma surgery. Neurosurgery.

[71] Stockhammer F, Misch M, Horn P, Koch A, Fonyuy N, Plotkin M (2009). Association of F18-fluoro-ethyl-tyrosin uptake and 5-aminolevulinic acid-induced fluorescence in gliomas. Acta Neurochir.

[72] Stummer W (2015). Response to journal club: 5-aminolevulinic acid-derived tumor fluorescence: the diagnostic accuracy of visible fluorescence qualities as corroborated by spectrometry and histology and postoperative imaging. Neurosurgery.

[73] Stummer W (2016). Commentary: combining 5-aminolevulinic acid fluorescence and intraoperative magnetic resonance imaging in glioblastoma surgery: a histology-based evaluation. Neurosurgery.

[74] Stummer W, Novotny A, Stepp H, Goetz C, Bise K, Reulen HJ (2000). Fluorescence-guided resection of glioblastoma multiforme by using 5-aminolevulinic acid-induced porphyrins: a prospective study in 52 consecutive patients. J Neurosurg.

[75] Stummer W, Pichlmeier U, Meinel T, Wiestler OD, Zanella F, Reulen HJ, Group AL-GS (2006). Fluorescence-guided surgery with 5-aminolevulinic acid for resection of malignant glioma: a randomised controlled multicentre phase III trial. Lancet Oncol.

[76] Stummer W, Stepp H, Moller G, Ehrhardt A, Leonhard M, Reulen HJ (1998). Technical principles for protoporphyrin-IX-fluorescence guided microsurgical resection of malignant glioma tissue. Acta Neurochir.

[77] Stummer W, Stocker S, Novotny A, Heimann A, Sauer O, Kempski O, Plesnila N, Wietzorrek J, Reulen HJ (1998). In vitro and in vivo porphyrin accumulation by C6 glioma cells after exposure to 5-aminolevulinic acid. J Photochem Photobiol B.

[78] Stummer W, Stocker S, Wagner S, Stepp H, Fritsch C, Goetz C, Goetz AE, Kiefmann R, Reulen HJ (1998). Intraoperative detection of malignant gliomas by 5-aminolevulinic acid-induced porphyrin fluorescence. Neurosurgery.

[79] Stummer W, Tonn JC, Goetz C, Ullrich W, Stepp H, Bink A, Pietsch T, Pichlmeier U (2014). 5-Aminolevulinic acid-derived tumor fluorescence: the diagnostic accuracy of visible fluorescence qualities as corroborated by spectrometry and histology and postoperative imaging. Neurosurgery.

[80] Stupp R, Mason WP, van den Bent MJ, Weller M, Fisher B, Taphoorn MJ, Belanger K, Brandes AA, Marosi C, Bogdahn U, Curschmann J, Janzer RC, Ludwin SK, Gorlia T, Allgeier A, Lacombe D, Cairncross JG, Eisenhauer E, Mirimanoff RO, European Organisation for R, Treatment of Cancer Brain T, Radiotherapy G, National Cancer Institute of Canada Clinical Trials G (2005). Radiotherapy plus concomitant and adjuvant temozolomide for glioblastoma. N Engl J Med.

[81] Talacchi A, Turazzi S, Locatelli F, Sala F, Beltramello A, Alessandrini F, Manganotti P, Lanteri P, Gambin R, Ganau M, Tramontano V, Santini B, Gerosa M (2010). Surgical treatment of high-grade gliomas in motor areas. The impact of different supportive technologies: a 171-patient series. J Neuro-Oncol.

[82] Teixidor P, Arraez MA, Villalba G, Garcia R, Tardaguila M, Gonzalez JJ, Rimbau J, Vidal X, Montane E (2016). Safety and efficacy of 5-aminolevulinic acid for high grade glioma in usual clinical practice: a prospective cohort study. PLoS One.

[83] Tsugu A, Ishizaka H, Mizokami Y, Osada T, Baba T, Yoshiyama M, Nishiyama J, Matsumae M (2011). Impact of the combination of 5-aminolevulinic acid-induced fluorescence with intraoperative magnetic resonance imaging-guided surgery for glioma. World Neurosurg.

[84] Watts C, Sanai N (2016). Surgical approaches for the gliomas. Handb Clin Neurol.

[85] Widhalm G, Kiesel B, Woehrer A, Traub-Weidinger T, Preusser M, Marosi C, Prayer D, Hainfellner JA, Knosp E, Wolfsberger S (2013). 5-Aminolevulinic acid induced fluorescence is a powerful intraoperative marker for precise histopathological grading of gliomas with non-significant contrast-enhancement. PLoS One.

[86] Widhalm G, Wolfsberger S, Minchev G, Woehrer A, Krssak M, Czech T, Prayer D, Asenbaum S, Hainfellner JA, Knosp E (2010). 5-Aminolevulinic acid is a promising marker for detection of anaplastic foci in diffusely infiltrating gliomas with nonsignificant contrast enhancement. Cancer.

[87] Wirtz CR, Knauth M, Staubert A, Bonsanto MM, Sartor K, Kunze S, Tronnier VM (2000). Clinical evaluation and follow-up results for intraoperative magnetic resonance imaging in neurosurgery. Neurosurgery.

[88] Yamada S, Muragaki Y, Maruyama T, Komori T, Okada Y (2015). Role of neurochemical navigation with 5-aminolevulinic acid during intraoperative MRI-guided resection of intracranial malignant gliomas. Clin Neurol Neurosurg.

[89] Yamahara T, Numa Y, Oishi T, Kawaguchi T, Seno T, Asai A, Kawamoto K (2010). Morphological and flow cytometric analysis of cell infiltration in glioblastoma: a comparison of autopsy brain and neuroimaging. Brain Tumor Pathol.

[90] Yordanova YN, Moritz-Gasser S, Duffau H (2011). Awake surgery for WHO grade II gliomas within “noneloquent” areas in the left dominant hemisphere: toward a “supratotal” resection. Clinical article. J Neurosurg.

